# Comparative chemical profiling, cholinesterase inhibitions and anti-radicals properties of essential oils from *Polygonum hydropiper* L: A Preliminary anti- Alzheimer’s study

**DOI:** 10.1186/s12944-015-0145-8

**Published:** 2015-11-04

**Authors:** Muhammad Ayaz, Muhammad Junaid, Farhat Ullah, Abdul Sadiq, Mir Azam Khan, Waqar Ahmad, Muhammad Raza Shah, Muhammad Imran, Sajjad Ahmad

**Affiliations:** Department of Pharmacy, University of Malakand, Khyber pakhtoonkhwa (KPK), 18000 Pakistan; H.E. J. Research Institute of Chemistry, International Center for Chemical and Biological Sciences, University of Karachi, Karachi, 75270 Pakistan

**Keywords:** *Polygonum hydropiper*, Essential oils, GC-MS, Anticholinesterase and antioxidant

## Abstract

**Background:**

Cholinesterase inhibition is a vital target for the development of novel and mechanism based inhibitors, owing to their role in the breakdown of acetylcholine (ACh) neurotransmitter to treat various neurological disorders including Alzheimer’s disease (AD). Similarly, free radicals are implicated in the progression of various diseases like neurodegenerative disorders. Due to lipid solubility and potential to easily cross blood brain barrier, this study was designed to investigate the anticholinesterase and antioxidant potentials of the standardized essential oils from the leaves and flowers of *Polygonum hydropiper*.

**Methods:**

Essential oils from the leaves (Ph.LO) and flowers (Ph.FO) of *P. hdropiper* were isolated using Clevenger apparatus. Oil samples were analyzed by GC-MS to identify major components and to attribute the antioxidant and anticholinesterase activity to specific components. Acetylcholinesterase (AChE) and butyrylcholinesterase (BChE) inhibitory potentials of the samples were determined following Ellman’s assay. Antioxidant assays were performed using 1,1-diphenyl,2-picrylhydrazyl (DPPH), 2,2-azinobis[3-ethylbenzthiazoline]-6-sulfonic acid (ABTS) and hydrogen peroxide (H_2_O_2_) free radical scavenging assays.

**Results:**

In the GC-MS analysis 141 and 122 compounds were indentified in Ph.LO and Ph.FO respectively. Caryophylene oxide (41.42 %) was the major component in Ph.FO while decahydronaphthalene (38.29 %) was prominent in Ph.LO. In AChE inhibition, Ph.LO and Ph.FO exhibited 87.00** and 79.66***% inhibitions at 1000 μg/ml with IC_50_ of 120 and 220 μg/ml respectively. The IC_50_ value for galanthamine was 15 μg/ml. In BChE inhibitory assay, Ph.LO and Ph.FO caused 82.66*** (IC_50_ 130 μg/ml) and 77.50***% (IC_50_ 225 μg/ml) inhibitions respectively at 1000 μg/ml concentration. In DPPH free radical scavenging assay, Ph.LO and Ph.FO exhibited IC_50_ of 20 and 200 μg/ml respectively. The calculated IC_50_s were 180 & 60 μg/ml for Ph.LO, and 45 & 50 μg/ml for Ph.FO in scavenging of ABTS and H_2_O_2_ free radicals respectively.

**Conclusions:**

In the current study, essential oils from leaves and flowers of *P. hydropiper* exhibited dose dependent anticholinesterase and antioxidant activities. Leaves essential oil were more effective and can be subjected to further *in-vitro* and *in-vivo* anti-Alzheimer’s studies.

**Electronic supplementary material:**

The online version of this article (doi:10.1186/s12944-015-0145-8) contains supplementary material, which is available to authorized users.

## Background

The cholinergic concept of Alzheimer’s disease (AD) was initially resulted from postmortem studies of the brain [1, 2], which ultimately led to the development of new drugs based on the inhibition of the key enzymes acetylcholinesterase (AChE) and butyrylcholinesterase (BChE) [3, 4]. Therapy with such drugs resulted in a significant improvement in cognitive functions and also hampered the progression of the disease [5–7]. Two cholinesterases, AChE encoded by gene on chromosome 7 and BChE encoded by gene on chromosome 3 occur in the human central nervous system (CNS) [8, 9]. These enzymes share about 65 % amino acid sequence homology even though coded on different genes [10]. In the human brain BChE mostly appears to have a neuroglial distribution, while AChE is principally located within cholinergic axons and in the neuronal cell bodies. Both enzymes are also present in neuritic plaques and tangles in AD patients [11, 12]. The ratio of cholinesterases in the human brain varies during the course of AD. A decline of 10–15 % in the activity of AChE in the hippocampus and cerebral cortex has been reported in advanced stages of the disease, whereas BChE activity increases by 40–90 % [11, 13]. These changes in the ratio of cholinesterases and variation in the level of the neurotransmitters in dementia must be considered in order to optimize the therapeutic balance between AChE and BChE inhibitions. This balance may be sustained via the selective or non-selective inhibition of the enzymes. A significant correlation between the inhibition of BChE activity in the cerebrospinal fluid (CSF), but not AChE, with an enhancement in cognitive performance in patients with mild to moderate AD after treatment with rivastigmine (non-specific inhibitor of cholinesterases) has been reported [14]. Experimental data also revealed that BChE specific inhibitors not only raise the levels of acetylcholine (ACh) in rats but also improve memory in elderly rats [9, 15]. These findings also signify that inhibition of BChE in addition to AChE may be vital in the treatment of Alzheimer’s type dementia.

A currently available drug like tacrine is observed to have severe side effects like liver transaminase elevations and gastrointestinal complainsts [16], and are only useful in mild type of AD [17]. Therefore, it is required to search new, safe and effective drug candidates. Natural products are potential sources of novel bioactive compounds and have an extensive history of therapeutic utility since the establishment of human era. Galanthamine, an anticholinestrase alkaloid was isolated from snowdrop, and is approved for the therapy of AD [18]. Research has been directed to study the biological effects of plants traditionally used as cholinesterase inhibitors [18, 19].

Free radicals including reactive oxygen species (ROS) are implicated in a variety of disorders including neuro-inflammation, gastritis, ischemic heart diseases, reperfusion injury of tissues and atherosclerosis [20, 21]. Free radicals generated during oxidation process are converted to non-radical forms by catalase and hydroperoxidase enzymes in living systems. But in case of excessive radical generation or depletion of human immune system natural antioxidants as free radical scavengers may be required [22]. In Alzheimer’s patients and aging brain, dysfunctional mitochondria generate free radicals, thus lead to oxidative stress followed by oxidative damage and pathological changes. β-amyloid (Aβ) is a powerful originator of reactive oxygen and nitrogen species which are primary initiators of oxidative harm thus effecting neural, microglial, cerebrovascular cells and tissues [23]. Currently, available synthetic antioxidants including gallic acid esters, tertiary butylated hydroquinone and butylated hydroxy toluene (BHT) are associated with adverse health consequences [24]. Numerous natural bioactive compounds have been shown to possess strong antioxidant potential which reveals that these compounds have the ability to scavenge free radicals inside the body and provide very low chances of adverse effects [25, 26].

Among plants which have been investigated for the treatment of neurodegenerative disorders, *Polygonum hydropiper* is one of the most numerous genuses in the family *Polygonaceae* which is abundant in North West of Pakistan. This plant has a long history of use in folk medicine as remedy for the treatment of a multiplicity of disorders including inflammation, rheumatoid arthritis, epilepsy, headache, colic pain, fever, chill, joint pain, oedema and infectious diseases [27–29]. It is also used as diuretic, CNS stimulant, anthelmintic, to treat insomnia, kidney diseases, hemorrhoids, hypertension and angina [30]. Other species of *Polygonaceae* family have been reported for their effectiveness in Parkinson’s disease [31], cerebral ischemia [32] and neuroprotective agents [33]. We recently reported the solvent extracts of *P. hydropiper* for antioxidant, anticholinestrase activities and its potential effectiveness to treat neurodegenerative disorders [29]. Volatile constituents of the essential oils from *P. hydropiper* are expected to readily cross the blood–brain barrier owing to their small molecular size and lipophilic nature. Their volatile nature may also facilitate their administration in the form of inhalation avoiding the alimentary canal with its attendant denaturing of active molecular species.

## Results

### GC-MS analysis of samples

**Fig. 1:**
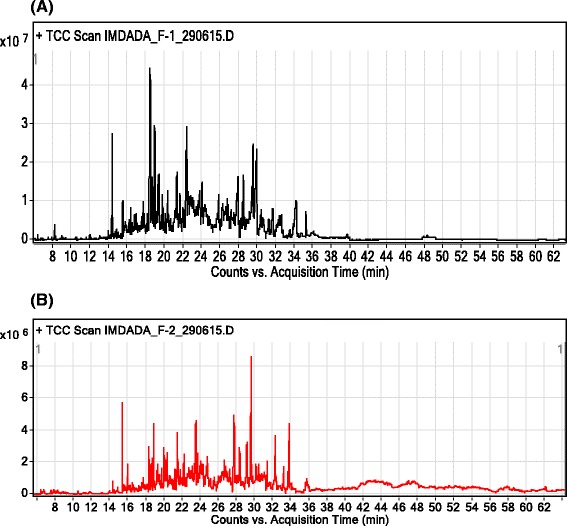
GC-MS chromatogram of essential oils from leaves (**a**) and flowers (**b**) of *Polygonum hydropiper*

**Fig. 2:**
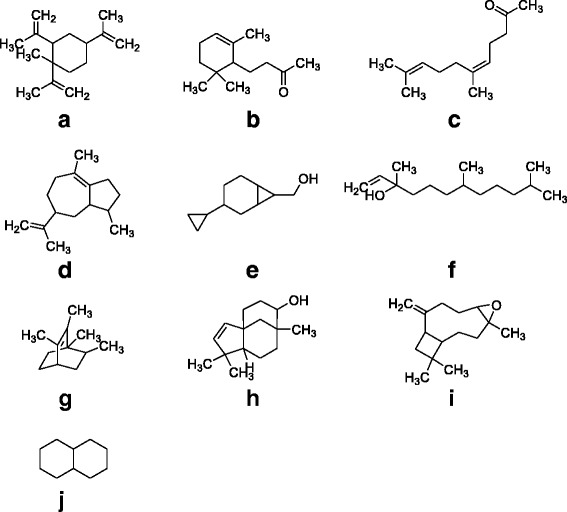
Major identified compounds in the GC-MS analysis of essential oils from leaves of *Polygonum hydropiper *(**a**) 1-Methyl-1,2,4-tri(prop-1-en-2-yl)cyclohexane (**b**) 4-(2,6,6-Trimethylcyclohex-2-enyl)butan-2-one (**c**) (Z)-6,10-dimethylundeca-5,9-dien-2-one (**d**) (Z)-1,2,3,5,6,7,8,8a-octahydro-1,4-dimethyl-7-(prop-1-en-2-yl)azulene (**e**) 3-Cyclopropylbicyclo[4.1.0]heptan-7-yl)methanol (**f**) 3,7,11-Trimethyldodec-1-en-3-ol (**g**) 1,2,3,6-Tetramethylbicyclo[2.2.2]oct-2-ene (**h**) (1R,5S,8R,9R)-4,4,8-trimethyltricyclo[6.3.1.0(1,5)]dodeca-2-en-9-ol (**i**) b-Caryophyllene epoxide (**j**) Decahydronaphthalene

**Fig. 3:**
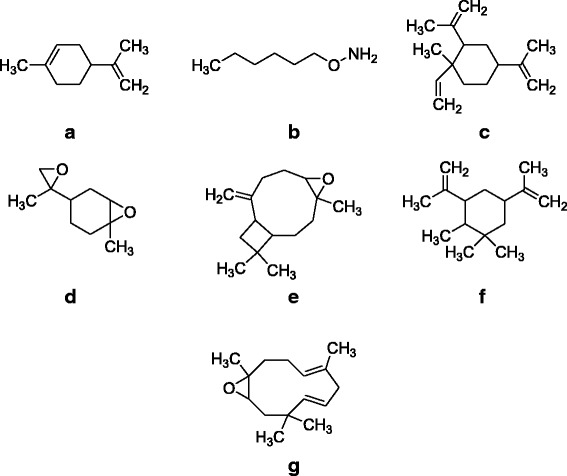
Major compounds identified in GC-MS analysis of essential oils from flower of *Polygonum hydropiper* (**a**) 1-methyl-4-(prop-1-en-2-yl)clohex-1-ene (**b**) O-Hexylhydroxylamine (**c**) 1-methyl-2,4-dl(prop-1-en-2-yl)-1-vinylcyclohexane (**d**) 1-methyl-4-(2-methyloiran-2-yl)-7-oxa-bicyclo(4.1.0)heptane (**e**) (-)-β-Caryophylleneepoxide (**f**) 1,1,2-trimethyl-3,5-di(prop-1-en-2-yl)cycloheaxane (**g**) (4E,7E)-1,5,9,9-tetramethyl-12-oxabicyclo[9.1.0]dodeca-4,7-diene

In GC, GC-MS analysis of Ph.LO, 141 compounds were identified (as shown in Fig. 1) among which decahydronaphthalene (38.29 %), bicyclo [2.2.2]oct-2-ene, 1,2,3,6-tetramethyl (36.33 %), β-elemene *cis*-1,3-diisopropenyl-*trans*-4-vinyl-4-methylcyclohexane (6.81 %), *cis*-geranylacetone (3.72 %), β-caryophyllene epoxide (2.69 %) were in higher concentrations as shown in Fig. 2, Table 1(A) (The details of all compounds identified in the leaf oil is shown in Additional file 1: Table S1 in supporting information). In analysis of Ph.FO, caryophylene oxide (41.42 %), beta caryophyllene epoxide (18.17 %), humulene oxide (16.09 %), β-elemene *cis*-1,3-diisopropenyl-*trans*-4-vinyl-4-methylcyclohexane (4.76 %), 3,5-diisopropenyl-1,1,2-trimethylcyclohexane (3.83 %) and limonene (1.79 %) were in high concentrations as given in Fig. 3, Table 1(B) (The details of all compounds identified in the leaf oil is shown in Additional file 2: Table S2 in supporting information).

**Table 1:** User Chromatogram peaks list for major compounds identified in essential oils from leaves (A) and flowers (B) of *Polygonum hydropiper*

RT	Height	Height %	Area	Area %	Area Sum %	Base Peak m/z	Width
A							
14.359	11039380	39.24	27045773	17.79	6.81	81.1	0.114
14.822	2441741	8.68	5347147	3.52	1.35	43.1	0.084
15.505	6158002	21.89	14751160	9.7	3.72	43.1	0.111
16.382	4049393	14.39	10136429	6.67	2.55	93.1	0.111
17.722	3399191	12.08	11251734	7.4	2.83	79.1	0.111
17.839	3697087	13.14	7865419	5.17	1.98	69.1	0.084
18.449	26630825	94.65	144220508	94.88	36.33	79.1	0.178
18.482	5054455	17.96	3502616	2.3	0.88	161.1	0.027
18.663	4507537	16.02	10670668	7.02	2.69	83	0.084
18.951	28135093	100	151997749	100	38.29	109.1	0.191
B							
6.353	239671	6.66	548139	4.31	1.79	68.1	0.087
7.853	124166	3.45	277247	2.18	0.9	43.1	0.094
14.33	719035	19.97	1461208	11.5	4.76	81.1	0.077
16.369	196811	5.47	404002	3.18	1.32	43.1	0.07
16.751	181702	5.05	370460	2.91	1.21	43.1	0.074
18.319	3E + 06	71.93	5575945	43.87	18.17	43.1	0.084
18.61	531433	14.76	1176145	9.25	3.83	83	0.084
18.821	2E + 06	50.77	4937507	38.84	16.09	43.1	0.124
19.97	959858	26.66	2359693	18.56	7.69	43.1	0.087
21.433	4E + 06	100	12711263	100	41.42	43.1	0.134

### Cholinesterase inhibition assays

The acetyl and butyrylcholinesterase inhibitions potentials of essential oils isolated from leaves and flowers of *P. hydropiper* are shown in Table 2.

**Table 2:** Results of AChE and BChE inhibitory activity of essential oils from leaves and flowers of *Polygonum hydropiper*

Sample	% AChE inhibition Mean ± SEM (*n* = 3)			% BChE inhibition Mean ± SEM (*n* = 3)		
	Concentration	% inhibition	IC_50_	Concentration	% inhibition	IC_50_
Essential oils from leaves (Ph.Lo)	12.5	28.00 ± 0.57***	120	12.5	20.66 ± 1.20***	130
	25	36.50 ± 0.44***		25	31.00 ± 1.15***	
	50	44.66 ± 1.20***		50	40.33 ± 0.88***	
	100	52.00 ± 1.52***		100	48.66 ± 1.20***	
	125	57.33 ± 0.88***		125	52.00 ± 0.57***	
	250	63.66 ± 0.33***		250	61.00 ± 1.15***	
	500	71.00 ± 0.57***		500	70.66 ± 0.88***	
	1000	87.00 ± 1.15**		1000	82.66 ± 1.20***	
Essential oils from flowers (Ph.Fo)	12.5	21.66 ± 0.66***	220	12.5	18.00 ± 0.57***	225
	25	28.00 ± 1.15***		25	23.00 ± 1.15***	
	50	35.00 ± 0.57***		50	27.33 ± 0.33***	
	100	43.33 ± 1.45***		100	35.00 ± 0.16***	
	125	49.00 ± 0.00***		125	46.66 ± 1.20***	
	250	55.00 ± 1.00***		250	53.66 ± 0.88***	
	500	61.66 ± 1.20***		500	64.00 ± 0.00***	
	1000	79.66 ± 0.88***		1000	77.50 ± 0.44***	
Galanthamine (P. Control)	12.5	51.00 ± 0.00	15	12.5	60.00 ± 0.57	10
	25	60.50 ± 0.44		25	70.00 ± 1.52	
	50	65.66 ± 0.33		50	77.66 ± 1.20	
	100	72.00 ± 2.64		100	81.00 ± 0.00	
	125	77.00 ± 0.00		125	86.66 ± 1.76	
	250	83.33 ± 0.66		250	89.33 ± 1.45	
	500	87.00 ± 1.20		500	92.33 ± 0.66	
	1000	92.33 ± 0.33		1000	96.00 ± 1.52	

Results were expressed as means ± S*.*E*.*M. The *P* values less than 0.05 were considered as statistically significant. Values significantly different when compared to slandered drug (Galanthamine) at the same concentration i.e. *: *P* < 0.05, **: *P* < 0.01 and ***: *P* < 0.001

#### Acetylcholinesterase (AChE) inhibition assay

In acetylcholinesterase (AChE) inhibition assay, essential oils from leaves (Ph.LO) exhibited 87.00 ± 1.15**, 71.00 ± 0.57***, 63.66 ± 0.33***, 57.33 ± 0.88***, 52.00 ± 1.52***, 44.66 ± 1.20***, 36.50 ± 0.44*** and 28.00 ± 0.57*** % inhibition at concentrations of 1000, 500, 250, 125, 100, 50, 25 and 12.50 μg/ml respectively. Likewise, essential oils from flowers (Ph.FO), showed 79.66 ± 0.88***, 61.66 ± 1.20*** and 55.00 ± 1.00***, 49.00 ± 0.00***, 43.33 ± 1.45***, 35.00 ± 0.57***, 28.00 ± 1.15*** and 21.66 ± 0.66***% inhibitions at concentrations of 1000, 500, 250, 125, 100, 50, 25 and 12.50 μg/ml respectively. Highest AChE inhibitions from standard drug galanthamine were 92.33 ± 0.33, 87.00 ± 1.20, 83.33 ± 0.66, 77.00 ± 0.00 and 72.00 ± 2.64 % at concentration of 1000, 500, 250, 125 and 100 μg/ml respectively. The IC_50_ for Ph.LO, Ph.FO and galanthamine were 120, 220 and 15 μg/ml respectively.

#### Butyrylcholinesterase (BChE) inhibition assay

In Butyrylcholinesterase (BChE) inhibition assay, highest Ph.LO exhibited 82.66 ± 1.20***, 70.66 ± 0.88***, 61.00 ± 1.15*** and 52.00 ± 0.57***, 48.66 ± 1.20***, 40.33 ± 0.88***, 31.00 ± 1.15*** and 20.66 ± 1.20***% enzyme inhibitions at concentrations of 1000, 500, 250, 125, 100, 50, 25 and 12.50 μg/ml respectively. Furthermore, Ph.FO showed 77.50 ± 0.44***, 64.00 ± 0.00***, 53.66 ± 0.88***, 46.66 ± 1.20***, 35.00 ± 0.16***, 27.33 ± 0.33***, 23.00 ± 1.15***, 18.00 ± 0.57***% BChE inhibitions at concentrations of 1000, 500, 250, 125, 100, 50, 25 and 12.50 μg/ml respectively. Galanthamine revealed 96.00 ± 1.52, 92.33 ± 0.66, 89.33 ± 1.45, 86.66 ± 1.76, 81.00 ± 0.00, 77.66 ± 1.20, 70.00 ± 1.52 and 60.00 ± 0.57 % inhibitions at the same concentrations. The IC_50_ calculated from dose response curve were 130, 225 and 10 μg/ml for Ph.LO, Ph.FO and galanthamine respectively.

### Antioxidant assays

The antioxidant potentials of essential oils from leaves and flowers of *P. hydropiper* were determined using DPPH, ABTS and H_2_O_2_ free radicals. The results are summarized in Table 3.

**Table 3:** Antioxidant Potential of essential oils from *Polygonum hydropiper* leaves and flowers

Samples	DPPH free radical scavenging			ABTS free radical scavenging		H_2_O_2_ free radical scavenging	
	Conc. μg/ml	% inhibition	IC_50_	% inhibition	IC_50_	% inhibition	IC_50_
Essential oils from leaves (Ph.LO)	12.5	37.95 ± 0.29***	20	9.66 ± 1.33^ns^	180	25.66 ± 0.66 ^ns^	60
	25	46.66 ± 0.72^ns^		13.00 ± 1.15 ^ns^		33.16 ± 1.01 ^ns^	
	50	53.50 ± 0.86^ns^		25.66 ± 2.18 ^ns^		42.50 ± 0.28 ^ns^	
	100	60.66 ± 0.92*		27.50 ± 0.28 ^ns^		47.50 ± 1.04 ^ns^	
	200	65.16 ± 0.60*		42.66 ± 0.92 ^ns^		58.00 ± 0.28 ^ns^	
	400	72.00 ± 1.04***		58.16 ± 1.09*		65.16 ± 1.96 ^ns^	
	800	79.50 ± 0.28***		73.16 ± 1.01***		70.66 ± 0.88 ^ns^	
	1000	85.00 ± 1.15**		89.00 ± 0.50***		79.00 ± 1.00***	
Essential oils from Flower (Ph.FO)	12.5	22.83 ± 0.72***	200	30.00 ± 0.00 ^ns^	45	21.83 ± 0.60 ^ns^	50
	25	28.00 ± 0.57***		38.50 ± 0.86 ^ns^		32.00 ± 2.30 ^ns^	
	50	35.83 ± 0.60***		45.16 ± 0.60 ^ns^		40.83 ± 0.92 ^ns^	
	100	42.33 ± 0.44***		51.33 ± 0.66 ^ns^		49.83 ± 0.44 ^ns^	
	200	47.33 ± 1.30***		66.00 ± 1.15 ^ns^		58.50 ± 0.76 ^ns^	
	400	54.83 ± 2.92***		72.00 ± 0.86 ^ns^		64.00 ± 0.00 ^ns^	
	800	70.16 ± 0.60***		78.66 ± 0.88***		71.66 ± 3.17*	
	1000	81.33 ± 0.72***		87.33 ± 1.76***		77.16 ± 0.44***	
Ascorbic Acid (Positive control)	12.5	45.00 ± 0.50	5	49.16 ± 0.60	10	46.66 ± 0.72	7
	25	47.33 ± 1.30		56.50 ± 1.04		55. 16 ± 0.60	
	50	54.88 ± 1.30		63.16 ± 1.01		63.00 ± 0.00	
	100	63.00 ± 1.15		70.00 ± 0.00		68.58 ± 0.69	
	200	68.36 ± 0.57		75.45 ± 0.65		71.44 ± 0.58	
	400	79.85 ± 2.24		81.37 ± 0.64		76.45 ± 0.77	
	800	87.08 ± 0.47		88.37 ± 0.54		84.36 ± 0.64	
	1000	91.90 ± 0.96		94.30 ± 0.61		89.37 ± 0.65	

Each value represent mean ± S*.*E*.*M of three independent experimental results. Values significantly different when compared to slandered drug (Ascorbic acid) at the same concentration i.e. *: *P* < 0.05, **: *P* < 0.01 and ***: *P* < 0.001. ns: Values not significantly different in comparison to P. control

#### DPPH assay

In DPPH free radicals scavenging assay, Ph.LO exhibited 85.00 ± 1.15**, 79.50 ± 0.28***, 72.00 ± 1.04***, 65.16 ± 0.60*, 60.66 ± 0.92* and 53.50 ± 0.86, 46.66 ± 0.72 and 37.95 ± 0.29***% inhibitions at concentrations of 1000, 800, 400, 200, 100, 50, 25 and 12.50 μg/ml respectively. For Ph.FO highest DPPH scavenging activities observed were, 81.33 ± 0.72***, 70.16 ± 0.60***, 54.83 ± 2.92*** at concentrations of 1000, 800 and 400 μg/ml respectively. The IC_50_ calculated from dose–response curve were 20 and 200 μg/ml for Ph.LO & Ph.FO respectively. Ascorbic acid demonstrated 91.90 ± 0.96, 87.08 ± 0.47, 79.85 ± 2.24, 68.36 ± 0.57 and 63.00 ± 1.15 % inhibitions at concentrations of 1000, 800, 400, 200 and 100 μg/ml respectively attaining an IC_50_ of 5 μg/ml.

#### ABTS assay

In ABTS free radicals scavenging assay, 89.00 ± 0.50***, 73.16 ± 1.01***, 58.16 ± 1.09*, 42.66 ± 0.92, 27.50 ± 0.28, 25.66 ± 2.18, 13.00 ± 1.15 and 9.66 ± 1.33 % activity was observed for Ph.LO at concentrations of 1000, 800 and 400, 200, 100, 50, 25 and 12.5 μg/ml respectively with IC_50_ of 180 μg/ml. Likewise, Ph.FO exhibited a dose dependent radicals scavenging activity of 87.33 ± 1.76***, 78.66 ± 0.88***, 72.00 ± 0.86, 66.00 ± 1.15, 51.33 ± 0.66, 45.16 ± 0.60, 38.50 ± 0.86 and 30.00 ± 0.00 % at 1000, 800 and 400 200, 100, 50, 25 and 12.5 μg/ml respectively. The IC_50_ for Ph.FO was 45 μg/ml. The standard drug ascorbic acid exhibited IC_50_ of 45 μg/ml at the same tested concentrations.

#### Hydrogen peroxide assay

In H_2_O_2_ radicals scavenging assay, 79.00 ± 1.00***, 70.66 ± 0.88, 65.16 ± 1.96 and 58.00 ± 0.28, 47.50 ± 1.04, 42.50 ± 0.28, 33.16 ± 1.01 and 25.66 ± 0.66 % scavenging effect was observed with Ph.LO at concentrations of 1000, 800, 400, 200, 100, 50, 25 and 12.5 μg/ml respectively. Whereas, Ph.FO showed 77.16 ± 0.44***, 71.66 ± 3.17*, 64.00 ± 0.00, 58.50 ± 0.76, 49.83 ± 0.44, 40.83 ± 0.92, 32.00 ± 2.30 and 21.83 ± 0.60 % inhibitions at concentrations of 1000, 800, 400, 200, 100, 50, 25 and 12.5 μg/ml respectively. The IC_50_s were 60 and 50 μg/ml for Ph.LO and Ph.FO respectively. In comparison, the standard drug ascorbic acid attain an IC_50_ of 7 μg/ml.

## Discussion

Steam distillation, subsequently GC/MS and GC/FID analysis were used to determine the chemical compositions of essential oils from the leaves and flowers of *P. hydropiper*. Chromatograms with the identified peaks (Fig. 1) as well as the chemical structures of the major identified compounds from leaves and flowers oils are shown in Figs. 2 and 3 respectively. In GC, GC-MS analysis of Ph.LO, 141 compounds were identified among which decahydronaphthalene (38.29 %) was in highest concentration (Fig. 2, Table 1(A)). Likewise, in analysis of Ph.FO, caryophylene oxide (41.42 %) was present in highest concentration as given in Fig. 3, Table 1(B). The number of identified compounds in Ph.LO were greater than Ph.FO and both anticholinesterase and antioxidant potentials of Ph.LO were observed to be higher than Ph.FO. This is not astonishing that the chemical composition of essential oils greatly depends upon the genetics, age, season and varies with environmental conditions of the plant [34]. Up to the best of our knowledge this is the most detailed report on the chemical composition of essential oils from *P. hydropiper*.

**Fig. 4:**
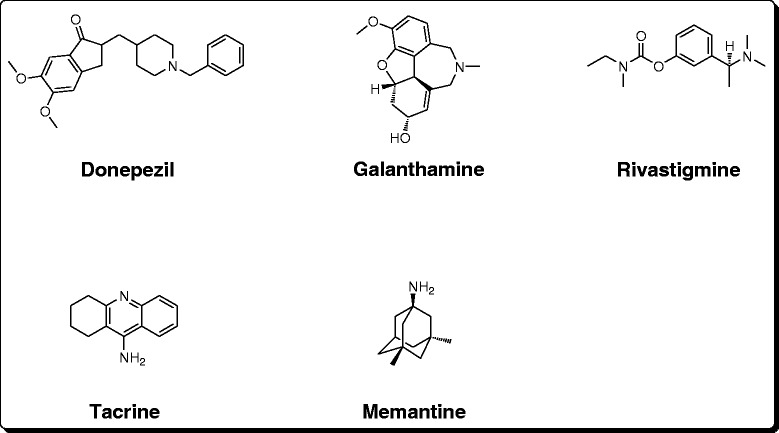
Clinically available drugs for Alzheimer’s therapy

Acetylcholinesterase (AChE) and butyrylcholinesterase (BChE) are the key enzymes catalyzing the breakdown of the important neurotransmitter acetylcholine (ACh) in the nervous system to form acetate and choline [25]. ACh insufficiency in the cerebral cortex of humans is among the vital pathophysiologies observed in AD patients [35, 36]. An important tool for treating AD is to boost the level of ACh in the brain by the administration of safe and effective AChE inhibitors [37]. Among the clinically approved drugs, four are cholinesterase inhibitors including tacrine, donepezil, rivastigmine and galantamine, whereas, the fifth one is glutamatergic system modifier called memantine (Fig. 4). Among these four drugs, the use of tacrine is limited due to hepatotoxic effects associated with it [38, 39]. Further, studies during clinical trials revealed that cholinesterase inhibitors may help AD patients to sustain their ability to perform routine activities with less frequent behavioral changes [40]. Other studies suggest that cholinesterase inhibitors may improve the cognitive performance of the AD patients even in the advanced stages of the disease [41]. Consequently, cholinesterase inhibitors may improve cognitive decline and thus reduce the emergence of new behavioral turbulence.

In the current study, we observed that Ph.LO were most effective against AChE causing 87.00 ± 1.15 % inhibition followed by Ph.FO with 79.66 ± 0.88 % enzyme inhibition at 1000 μg/ml. Among both oils, Ph.LO was more potent with IC_50_ of 120 μg/ml, while the IC_50_ for Ph.FO was 220 μg/ml. The IC_50_ value for galanthamine was 15 μg/ml. Both oils exhibited concentration dependent activity as shown in Table 2. In BChE inhibitory assay, again Ph.LO was more active causing 82.66 ± 1.20 % inhibition at 1000 μg/ml and IC_50_ of 130 μg/ml. Moreover, Ph.FO revealed 77.50 ± 0.44 % inhibition at the same concentration with IC_50_ of 225 μg/ml. Positive control inhibition was 96.00 ± 1.52 % at the same concentration and IC_50_ was 10 μg/ml. Presently, there is no complete preventative or curative drug therapy available for AD, leaving the symptomatic relief presented by AChE/BChE inhibitors as the single approved therapeutic choice. Recently, galanthamine from *Amaryllidaceae* family is approved for clinical use and has become a vital therapeutic option effective to retard the process of neurological degeneration in AD. Galanthamine provides an efficient symptomatic therapy for AD patients and also delay the progression of the disease. Another isoquinoline alkaloid berberine, isolated from *Rhizoma coptidis* and *Cortex phellodendri* is reported as an effective neuroprotective agent in diseases like cerebral ischemia, schizophrenia, AD, depression and anxiety [42, 43]. Berberine is reported to reduce extracellular Aβ fabrication and BACE activity without affecting the release of LDH in H4 neuroglioma (APPNL-H4) cells [8]. Berberine therapy also reduces cognitive dysfunction as indicated by decrease in errors using MWM task in comparison to usual reference memory and memory retention (probe trial) in APP transgenic mice. [44]. The essential oils in the current study exhibited comparative percent inhibitions with the standard drug in both assays. However, the IC_50_ of essential oils were higher than that of an orally administered standard drug galanthamine. We speculate that the essential oils administered in the form of vapors (aerosol) will have better availability than orally administered drugs due to high lipid solubility and bypassing pre-systemic metabolism. However, further in-vivo studies on genetically modified animals’ models are required to confirm its in-vivo bioavailability and potential efficacy in neurological disorders.

Modern research revealed that beta secretase enzyme (BACE1) catalyze the breakdown of amyloid precursor protein (APP) to form ß-amyloid peptides in AD brain, which provoke inflammatory process with consequent release of free radicals oxygen species causing neuronal damage [45–47]. Antioxidant drugs may contribute to AD chemotherapy by attenuation of the inflammatory pathways via scavenging of free radicals [37]. In recent times, natural products has got more attention as antioxidants as they are safer and these substances could be supplied as food components or in the form of pharmaceuticals for human use [48]. Among those, essential oils from aromatic and medicinal plants are well known to reveal antioxidant and cholinesterase inhibitory properties and thus can be very helpful in the treatment of AD [49]. In DPPH assay, Ph.LO was most effective causing 85.00 ± 1.15, 79.50 ± 0.28 and 72.00 ± 1.04 % free radicals scavenging at concentrations of 1000, 800 and 400 μg/ml respectively. DPPH free radicals scavenging activity of Ph.FO was 81.33 ± 0.72, 70.16 ± 0.60 and 54.83 ± 2.92 % at 1000, 800 and 400 μg/ml respectively. Ph.LO and Ph.FO exhibited IC_50_ of 20 and 200 μg/ml respectively, whereas, the IC_50_ of ascorbic acid was 5 μg/ml (Table 3). Likewise, In ABTS assay Ph.LO demonstrated 89.00 ± 0.50 % inhibitions of free radicals followed by Ph.FO with 87.33 ± 1.76 % inhibition at 1000 μg/ml. All fractions showed concentration dependent activity. For both samples, the IC_50_ values were 180 and 45 μg/ml respectively. In H_2_O_2_ free radicals scavenging assay, 79.00 ± 1.00 and 77.16 ± 0.44 % inhibitions were observed for Ph.LO and Ph.FO respectively at 1000 μg/ml. For these samples the IC_50_ were 60 and 50 μg/ml. Ph.LO was observed to be more effective against all tested free radicals. In both assays Ph.LO was more effective in comparison to Ph.FO which can be attributed to the presence of active compounds in Ph.LO. In the current study, the percent inhibitions of essential oils were comparative to standard drugs at the same concentrations. Though the IC_50_ of oils were higher than standard drugs, still the dual efficacy (anticholinesterase and antioxidant) of essential oils demonstrate their potential effectiveness in neurological disorders.

## Conclusions

Essential oils from *P. hydropiper* were investigated for the first time for anticholinesterase and antioxidant potentials. All samples exhibited concentration dependent enzyme inhibitions and anti-radical activities with Ph.LO most affective. In GC, GC-MS analysis 144 and 122 compounds were identified in Ph.LO and Ph.FO respectively. Further in-vivo studies are required for possible use of these samples in neurodegenerative disorders.

## Material and methods

### Collection of plant material

Fresh leaves of *P. hydropiper* were collected from Talash Valley Dir (L) Pakistan in the month of September 2014. The leaves were washed with distilled water to remove any dust. Whereas, flowers of *P. hydropiper* were in full bloom in the month of September 2014, in Talash valley Dir lower Pakistan and were collected. Plant samples were deposited at the herbarium, University of Malakand Chakdara (Dir), Pakistan with voucher no (H.UOM.BG.107).

### Isolation of the essential oils

Fresh leaves of *P. hydropiper* were macerated and hydrodistilled using a Clevenger type apparatus supplied with condenser. Distillation process was continued for 3 days at 100 °C, and the volatile oils (yellowish in color) were collected in glass bottles. Anhydrous sodium sulfate was used to remove water after extraction [50]. Flowers of *P. hydropiper* were hydro-distilled with a Likens–Nickerson-type apparatus using diethyl ether for 3 h. White to yellow color obtained which was dried over anhydrous sodium sulphate. Finally, the oils were properly sealed in glass vials and stored in refrigerator at −30 °C before further analysis.

### Gas chromatography (GC) analysis

Essential oils samples were analyzed by means of an Agilent USB-393752 gas chromatograph (Agilent Technologies, Palo Alto, CA, USA) with HHP-5MS 5 % phenylmethylsiloxane capillary column (30 m × 0.25 mm × 0.25 μm film thickness; Restek, Bellefonte, PA) equipped with an FID detector. The temperature of Oven was maintained at 70 °C for 1 min at first, and then increased at the rate of 6 °C/min to 180 °C for 5 min and lastly at the rate of 5 °C/min to 280 °C for 20 min. Injector and detector temperatures were set at 220 °C and 290 °C, correspondingly. Helium was used as carrier gas at a flow rate of 1 ml/min, and diluted samples (1/1000 in *n*-pentane, *v/v*) of 1.0 μl were injected manually in the split-less mode.

### Gas chromatography–mass spectrometry (GC/MS) analysis

GC/MS analysis of the oil samples were processed using an Agilent USB-393752 gas chromatograph (Agilent Technologies, Palo Alto, CA, USA) with a HHP-5MS 5 % phenylmethylsiloxane capillary column (30 m × 0.25 mm × 0.25 μm film thickness; Restek, Bellefonte, PA) outfitted with an Agilent HP-5973 mass selective detector in the electron impact mode (Ionization energy: 70 eV) working under the same experimental conditions as described for GC.

### Identification of components

Oils major constituents were recognized by comparison of their retention times with those of authentic compounds in the literature. Moreover, identification was done via the spectral data obtained from the Wiley and NIST libraries, as well as comparisons of the fragmentation pattern of the mass spectra with data published in the literature [51, 52] or with mass spectra from literature.

### Chemical and drugs

For cholinesterase inhibition assay, AChE from *Electric eel* (type-VI-S, code 1001596210) and BChE from equine serum Lyophilized (code 101292670) were purchased from Sigma-Aldrich GmbH USA. Enzyme substrates including acetylthiocholine iodide (code 101303874) and butyrylthiocholine Iodide (code 101334643) were purchased from Sigma-Aldrich UK and Sigma-Aldrich Switzerland respectively. Indicator substance, 5,5-dithio-bis-nitrobenzoic acid (DTNB) code 101261619 was purchased from Sigma-Aldrich Germany. Standard drug galanthamine hydrobromide *Lycoris* Sp. (code G1660) was purchased from Sigma-Aldrich France. For antioxidant assays, DPPH (code 101341986 Sigma Aldrich CHEMIE GmbH USA), ABTS (code 1001551916 Sigma Aldrich USA), K_2_S_2_O_4_ (Riedel-de Haen Germany) and Folin Ciocalteu reagent (FCR) were acquired from Merck Co. (Germany). Buffer system including (K_2_HPO_4_), (KH2PO_4_), KOH and solvents used were of extra pure quality.

### Anticholinesterase assays

AChE and BChE inhibitory potentials of the samples were carried out following Ellman’s assay [53, 54]. Using this procedure, acetylthiocholine iodide or butyrylthiocholine iodide are hydrolyzed by the respective enzymes to form 5-thio-2-nitrobenzoate anion which then form complex with DTNB and give UV detectable yellow color compound.

#### Preparation of solutions

Oil samples were dissolved in phosphate buffer (0.1 M) in concentrations of 12.5, 25, 50, 100, 125, 500 and 1000 μg/ml. Phosphate buffer (0.1 M with 8.0 ± 0.1 pH) was prepared by mixing K_2_HPO_4_ (17.4 g/L) and KH2PO_4_ (13.6 g/L) solution in a ratio of 94 % and 6 % respectively. pH was adjusted using potassium hydroxide. To prepare enzyme solutions, AChE (518U/mg solid) and BChE (7-16U/mg) were diluted in buffer solution (pH 8.0) up to final concentrations of 0.03 U/ml and 0.01 U/ml. Substrate solutions including ATchI, BTchI (0.0005 M) and DTNB (0.0002273 M), were prepared using distilled water and were refrigerated at 8 °C until use. Standard drug (galanthamine) dilutions were made in methanol.

#### Spectroscopic analysis

In each experiment, 5 μl enzyme solutions were added to the cuvette and oil samples were added at the above mentioned concentrations. Finally, DTNB reagent (5 μl) was added to the cuvette and the resultant mixture was incubated at 30 °C for 15 min using water bath. A substrate solution (5 μl) was added at the end and absorbance was measured at 412 nm using a double beam spectrophotometer (Thermo electron corporation USA). Negative control contained all components except oil samples, while positive control galanthamine (10 μg/ml) was used in the assay as standard cholinesterase inhibitor. Change in absorbance along with the reaction time was recorded for 4 h at 30 °C. The experiments were performed in triplicate. Enzymes activity and enzyme inhibition by control and tested samples were determined from the rate of absorption with change in time (*V* = ΔAbs /Δt) as; Enzyme inhibition (%) = 100 - percent enzyme activity;

$$ \mathrm{Enzyme}\ \mathrm{activity}\left(\%\right)=\frac{100 \times \mathrm{V}}{{\mathrm{V}}_{\max }} $$

Where (V_max_) is enzyme activity in the absence of inhibitor drug.

### Antioxidant assays

#### DPPH free radicals scavenging assay

Free radicals scavenging ability of the essential oil was determined following well established procedures [29, 55]. Different dilutions (12.5, 50, 100, 200, 400, 800 and 1000 μg/ml) of essential oils (0.1 ml) were added to 0.004 % methanolic solution of DPPH. After 30 min, absorbance was measured at 517 nm using UV spectrophotometer (Thermo electron corporation, USA). Percent DPPH scavenging activity was calculated as;

$$ \frac{{\mathrm{A}}_0-{\mathrm{A}}_1}{{\mathrm{A}}_0}\times 100 $$

Ascorbic acid was used as positive control. Where A_0_ characterize absorbance of control and A_1_ is the absorbance of the essential oils. All experiments were performed in triplicate and inhibition graphs were made with the help of GraphPad prism program (GraphPAD, San Diego, California, USA). Median inhibitory concentrations IC_50_ values were calculated using Microsoft Excel programme.

#### ABTS free radicals scavenging assay

The ABTS free radical scavenging potential of samples were evaluated using previously reported procedure [56]. The test is based on the ability of antioxidants present in the sample to scavenge ABTS radicals leading to reduction. Using this procedure, solutions of ABTS 7 mM and potassium persulphate (K_2_S_2_O_4_) 2.45 mM were mixed and stored in dark place at room temperature for 12–16 h to obtain a dark colored solution. This solution was diluted using Phosphate buffer (0.01 M) pH 7.4 and absorbance value was adjusted to 0.70 at 734 nm. Finally, 300 μl solution of test sample was added to 3.0 ml of ABTS solution in cuvette and was analyzed spectrophotometerically at 734 nm. The decline in absorbance was determined after one minute of mixing the solutions and analysis was continued for 6 min. Ascorbic acid was used as positive control. The assay was repeated in triplicate and percentage inhibition was calculated using formula:

$$ \%\ \mathrm{scavenging}\ \mathrm{effect} = \mathrm{control}\ \mathrm{absorbance}\ \hbox{-}\ \mathrm{sample}\ \mathrm{absorbance}\ /\mathrm{control}\ \mathrm{absorbance} \times 100 $$

#### Hydrogen peroxide free radicals scavenging assay

The hydrogen peroxide scavenging activity of extracts was determined using methods described previously [57]. Using this method 2 mM hydrogen peroxide solution was prepared in 50 mM phosphate buffer having pH of 7.4. Oil samples (0.1 ml) were taken in test tubes and their volumes were increased to 0.4 ml using 50 mM phosphate buffer solution. Hydrogen peroxide (0.6 ml) was added to the tubes and was vertexed. Absorbance of each sample was measured at 230 nm against the blank after 10 min. Hydrogen peroxide scavenging activity was calculated using equation;

$$ \frac{1-\mathrm{absorbance}\ \mathrm{of}\ \mathrm{sample}}{\mathrm{Absorbance}\ \mathrm{of}\ \mathrm{control}}\times 100 $$

### Estimation of IC_50_ values

Concentrations of the oils which inhibited substrate hydrolysis (AChE and BChE) by 50 % (IC_50_) were calculated from dose response ratio using Microsoft Excel program. In DPPH, ABTS and H_2_O_2_ the IC_50_ values were calculated using the same procedure.

### Statistical data analysis

All the assays were repeated in triplicate and values were expressed as mean ± S*.*E*.*M. One way ANOVA followed by Dunnett’s multiple comparison test was applied for the comparison of positive control with the test group at 95 % confidence interval using GraphPad prism Software USA. The *P* values less than 0.05 were considered as statistically significant.

## Additional files

Additional file 1: Table S1. Details of compounds identified in GC, GC-MS analysis of essential oils from leaves of *Polygonum hydropiper*. (DOCX 29 kb) Additional file 2: Table S2. Details of compounds identified in GC, GC-MS analysis of essential oils from flowers of *Polygonum hydropiper*. (DOCX 28 kb)
