# Supplementary material for: Comparative chemical profiling, cholinesterase inhibitions and anti-radicals properties of essential oils from Polygonum hydropiper L: A Preliminary anti- Alzheimer’s study
Source: Lipids Health Dis. 2015 Nov 4;14:141. doi: 10.1186/s12944-015-0145-8 (PMC4632677; doi:10.1186/s12944-015-0145-8)
Supplement: Additional file 1: Table S1. — Details of compounds identified in GC, GC-MS analysis of essential oils from leaves of Polygonum hydropiper. (DOCX 29 kb) [file 12944_2015_145_MOESM1_ESM.docx]

**Additional file 1: Table S1:** Detail of compounds identified in GC, GC-MS analysis of essential oils from leaves of *P. hydropiper*.

| Compound Chemical / Label Name | Common Name | RT | Formula | Hits (DB) |
| --- | --- | --- | --- | --- |
| Cpd 1:(1E)-1-(Pentyloxy)-1-butene | NF | 6.059 | C9H18O | 10 |
| Cpd 2: Cyclohexene, 1-methyl-4-(1-methylethenyl) | Limonene | 6.351 | C10H16 | 10 |
| Cpd 3: 5-Oxopentanoic acid | ketopentanoate | 6.903 | C5H8O3 | 10 |
| Cpd 4: N-Formyl-2-hydroxy-3-methyl-2-(1-methylethyl) butanamide | NF | 7.519 | C9H17NO3 | 10 |
| Cpd 5: 4,4-dimethyl-1-heptene | NF | 7.851 | C9H18 | 10 |
| Cpd 6: 6-Methyl-2,4-heptanedione | Isovalerylacetone | 8.222 | C8H14O2 | 10 |
| Cpd 7: (5E)-3,6-Dimethyl-5-octen-2-one | NF | 8.272 | C10H18O | 10 |
| Cpd 8: 6-Methyl-5-octen-2-one | NF | 8.819 | C9H16O | 10 |
| Cpd 9: 1,3,3-trimethyl-2-norbornanol | Fenchol | 8.973 | C10H18O | 10 |
| Cpd 10: trans-2,7-Dimethyl-4,6-octadien-2-ol | NF | 9.061 | C10H18O | 10 |
| Cpd 11: 3-Chloro-2-methyl-2-pentanol | NF | 9.183 | C6H13ClO | 10 |
| Cpd 12: Octanoic acid | Caprylic acid | 9.344 | C8H16O2 | 10 |
| Cpd 13:6-Methyl-1-heptanol | Isooctanol | 9.917 | C8H18O | 10 |
| Cpd 14: Ethoxymethoxy cyclohexane | NF | 10.45 | C9H18O2 | 10 |
| Cpd 15: 2-Ethyl-1-butanol, trifluoroacetate | NF | 10.516 | C8H13F3O2 | 10 |
| Cpd 16: 1-(2,2-Dimethylcyclopentyl)ethanone | NF | 10.924 | C9H16O | 10 |
| Cpd 17: cis-13-Octadecenal | NF | 11.116 | C18H34O | 10 |
| Cpd 18: Octane, 1-cyclopropyl | Octylcyclopropane | 11.572 | C11H22 | 10 |
| Cpd 19: (2S,4aS.8aR)-(-)-5,5,8a-Trimethyldecahydronaphthalene-2-yl acetate | NF | 11.947 | C15H26O2 | 10 |
| Cpd 20: Bornyl acetate / Borneol acetate | Endobornyl Acetate | 11.993 | C12H20O2 | 10 |
| Cpd 21: 6-Methyl-5-octen-2-one | NF | 12.131 | C9H16O | 10 |
| Cpd 22: Octanal, 7-hydroxy-3,7-dimethyl | Hydroxycitronellal/ Fixol | 12.631 | C10H20O2 | 10 |
| Cpd 23: 5,9-Tetradecadiyne | NF | 12.999 | C14H22 | 10 |
| Cpd 24: 2-Isopropenyl-5-methyl-6-hepten-1-ol | NF | 13.455 | C11H20O | 10 |
| Cpd 25: Limonene Dioxide -2 | Limonene Dioxide-2 | 13.767 | C10H16O2 | 10 |
| Cpd 26: 2-Pentanone, 3-[(acetyloxy)methyl]-3,4-dimethyl-, (.+-.)- | NF | 13.953 | C10H18O3 | 10 |
| Cpd 27: Bicyclo[2.2.1]heptane-2-carboxaldehyde, 3-methyl-, (2-endo,3-exo)- | Limonene Dioxide-1 | 14.091 | C9H14O | 10 |
| Cpd 28: Limonene Dioxide 1 | NF | 14.201 | C10H16O2 | 10 |
| Cpd 29: CIS-1,3-Diisopropenyl-Trans-4-Vinyl-4-Methylcyclohexane | beta.-Elemene | 14.359 | C15H24 | 10 |
| Cpd 30: 2-Butanone, 4-(2,2-dimethyl-6-methylenecyclohexyl) | NF | 14.446 | C13H22O | 10 |
| Cpd 31: 4-Methylhept-6-En-3-One | NF | 14.535 | C8H14O | 10 |
| Cpd 32: Gamma Caryophyllene | isocaryophyllene | 14.708 | C15H24 | 10 |
| Cpd 33: 2-Butanone, 4-(2,6,6-trimethyl-2-cyclohexen-1-yl) | Dihydro .alpha. ionone | 14.822 | C13H22O | 10 |
| Cpd 34: Igamma Caryophyllene | isocaryophyllene | 14.988 | C15H24 | 10 |
| Cpd 35:p-Menth-8-en-1-ol, stereoisomer | cis-.beta.-Terpineol | 15.092 | C10H18O | 10 |
| Cpd 36:7,8-Dihydro beta ionone | Dihydro beta ionone | 15.277 | C13H22O | 10 |
| Cpd 37: 5,9-Undecadien-2-one, 6,10-dimethyl | cis-Geranylacetone | 15.504 | C13H22O | 10 |
| Cpd 38: 4-Isopropenyl-1-methyl-7-oxabicyclo[4.1.0]heptan-2-one | cis-Carvone oxide | 15.648 | C10H14O2 | 10 |
| Cpd 39: 1,3,6-Octatriene, 3,7-Dimethyl-, (E)- | Beta-Ocimene | 15.697 | C10H16 | 10 |
| Cpd 40: (E)-3,4-Epoxy-1-(1',2'-epoxy-3',3'-epoxymethano-2',6',6'-trimethyl-1'-cycloh... | NF | 15.751 | C15H22O3 | 10 |
| Cpd 41: Cyclopentane, 2-ethylidene-1,1-dimethyl | NF | 15.843 | C9H16 | 10 |
| Cpd 42: Trans-Sabinene Hydrate | 4-Thujanol | 15.901 | C10H18O | 10 |
| Cpd 43:(-)-Globulol | Globulol | 16.105 | C15H26O | 10 |
| Cpd 44: 2-Heptene, 2-methyl-6-p-tolyl | NF | 16.185 | C15H22 | 10 |
| Cpd 45: 1,6,10-Dodecatrien-3-ol, 3,7,11-trimethyl | Nerolidol/ E-farnesol | 16.27 | C15H26O | 10 |
| Cpd 46: Guaia-1(10),11-diene | Alpha Bulnesene | 16.382 | C15H24 | 10 |
| Cpd 47: 2(3H)-Benzofuranone, hexahydro-4,4,7a-trimethyl | Tetrahydroactinidiolide | 16.542 | C11H18O2 | 10 |
| Cpd 48: 1.Xi.,6.xi.,7.xi.-Cadina-4,9-diene | Alpha Muurolene | 16.591 | C15H24 | 10 |
| Cpd 49: Alpha-Bisabolene | Bisabolene | 16.707 | C15H24 | 10 |
| Cpd 50: 4-Isopropenyl-1-methyl-7-oxabicyclo[4.1.0]heptane | cis Limonene oxide | 16.768 | C10H16O | 10 |
| Cpd 51: (+)-Sativen | Sativene | 16.84 | C15H24 | 10 |
| Cpd 52: 4-Isopropenyl-1-methylcyclohexanol | Terpineol | 16.958 | C10H18O | 10 |
| Cpd 54: Farnesyl Acetone C | NF | 17.017 | C18H30O | 3 |
| Cpd 55: 2(1H)-Naphthalenone, octahydro-4a,7,7-trimethyl-, ciss | NF | 17.205 | C13H22O | 10 |
| Cpd 56: Allyl(chloromethyl) dimethylsilane | NF | 17.363 | C6H13ClSi | 4 |
| Cpd 57: 3-Allyl-2,6,6-trimethylbicyclo[3.1.1]heptane | NF | 17.413 | C13H22 | 10 |
| Cpd 58: 1,13-Tetradecadiene | NF | 17.594 | C14H26 | 10 |
| Cpd 59: Bicyclo[4.1.0]heptane,-3-cyclopropyl,-7-hydroxymethyl, trans | NF | 17.72 | C11H18O | 10 |
| Cpd 60: cis-5-Methyl-2,3,3a,4,7,7a-hexahydroinden-1-one | NF | 17.757 | C10H14O | 8 |
| Cpd 61: 1,6,10-Dodecatrien-3-ol, 3,7,11-trimethyl-, [S-(Z)]- $$ (+)-Nerolidol | NF | 17.838 | C15H26O | 10 |
| Cpd 62: 2H-cyclopropa[g]benzofuran, 4,5,5a,6,6a,6b-hexahydro-4,4,6b-Trimethyl | Benzofuran | 17.926 | C15H22O | 7 |
| Cpd 63: Cyclopropanemethanol, 2-methyl-2-(4-methyl-3-pentenyl) | NF | 18.07 | C11H20O | 10 |
| Cpd 64: Cyclohexanol, 2-Methylene-5-(1-Methylethenyl)-, (1s-Trans) | NF | 18.205 | C10H16O | 10 |
| Cpd 65: Bicyclo[2.2.2]oct-2-ene, 1,2,3,6-tetramethyl | NF | 18.448 | C12H20 | 10 |
| Cpd 66: (1R,5S,8R,9R)-4,4,8-trimethyltricyclo[6.3.1.0(1,5)]dodeca-2-en-9-ol | NF | 18.481 | C15H24O | 10 |
| Cpd 67: Limonene Dioxide 2 | Limonene Dioxide 2 | 18.539 | C10H16O2 | 10 |
| Cpd 68: 3-(1-Methylhept-1-enyl)-5-methyl-2,5-dihydrofuran-2-one | NF | 18.594 | C13H20O2 | 10 |
| Cpd 69:(-)-.beta.-Caryophyllene epoxide | Caryophyllene oxide | 18.662 | C15H24O | 10 |
| Cpd 70:1,1,4,7-Tetramethyldecahydro-1H-cyclopropa[e]azulen-4-ol | Epiglobulol | 18.747 | C15H26O | 10 |
| Cpd 71: 1-Dodecyn-3-ol | NF | 18.931 | C12H22O | 1 |
| Cpd 72: Naphthalene, decahydro | Decahydronaphthalene | 18.951 | C10H18 | 10 |
| Cpd 73: 7,8-Epoxy-.alpha.-ionone | NF | 19.056 | C13H20O2 | 10 |
| Cpd 74: 3-Heptadecen-5-yne, (Z)- | NF | 19.162 | C17H30 | 10 |
| Cpd 75: Geranyl acetate [trans-3,7-dimethyl-2,6-octadien-1-ylacetate] | NF | 19.271 | C12H20O2 | 3 |
| Cpd 76: cis-3-Hexenyl phenyl acetate | Benzeneacetic acid | 19.316 | C14H18O2 | 10 |
| Cpd 77: (-)-Caryophyllene oxide | Caryophyllene epoxide | 19.393 | C15H24O | 10 |
| Cpd 78: 7,8,8a,9-Tetrahydro-8a-methylnaphtho[2,3-b]furan-4,5(4aH,6H)-dione | NF | 19.427 | C13H14O3 | 10 |
| Cpd 79: Veridiflorol | Veridiflorol | 19.519 | C15H26O | 10 |
| Cpd 80: cyclohexane, 1,5-diethenyl-2,3-dimethyl-, (1.alpha.,2.beta.,3.beta.,5.beta.)- | NF | 19.784 | C12H20 | 10 |
| Cpd 81: 1-(3,3-dimethyl-bicyclo[2.2.1.]hept-2-yl)-2-penten-1-one | NF | 19.973 | C14H22O | 1 |
| Cpd 82: 2,6,10,14-Tetramethyl-2R,3R-epoxy-6E,10E,14E-hexadecatrien-1,16-diol | NF | 20.095 | C20H34O3 | 10 |
| Cpd 83: 8(12)-Drimen-7.alpha.,11-diol | NF | 20.126 | C15H26O2 | 10 |
| Cpd 84: 6-exo-Methoxy-3-methyl-3a,6a-dihydrofuro[3,4-d]isoxazol-4(6H)-one | NF | 20.215 | C7H9NO4 | 1 |
| Cpd 85: 11-Hexadecyn-1-ol | NF | 20.338 | C16H30O | 10 |
| Cpd 86: 2,6-Dimethyl-4-oxo-11-oxatricyclo[5.4.2.1(7,10).0(5,10)]undec-8-ene | NF | 20.381 | C12H16O2 | 5 |
| Cpd 87: 1,3,6,10-Dodecatetraene, 3,7,11-trimethyl- | Farnesene | 20.669 | C15H24 | 10 |
| Cpd 88: Bergamotol, Z-.alpha.-trans- | Bergamotol | 20.826 | C15H24O | 10 |
| Cpd 89: Pyrimidine, 4-cyclopropyl- | Cyclopropyl pyrimidine | 20.909 | C7H8N2 | 6 |
| Cpd 90: 7-Oxabicyclo[4.1.0]heptane, 1-methyl-4-(2-methyloxiranyl)- | Dipentene dioxide | 21.338 | C10H16O2 | 10 |
| Cpd 91: 5-Methylenedec-9-en-2-one | NF | 21.645 | C11H18O | 10 |
| Cpd 92: Bi-1-cycloocten-1-yl | NF | 21.883 | C16H26 | 10 |
| Cpd 93: Limonene Dioxide 2 | Limonene Dioxide 2 | 21.966 | C10H16O2 | 10 |
| Cpd 94: Limonene Dioxide 4 | Limonene Dioxide 4 | 22.123 | C10H16O2 | 10 |
| Cpd 95:Benzene, 4-fluoro-1,2-dimethyl- | 4-fluoro-1,2-xylene | 22.383 | C8H9F | 10 |
| Cpd 96: 1-Pentyn-3-one, 4-methyl- | 2-Methyl-3-Oxo-4-Pentyne | 22.419 | C6H8O | 1 |
| Cpd 97: Methyl 10,12-heptadecadiynoate | NF | 22.507 | C18H28O2 | 10 |
| Cpd 98: 2,6,10,14,18,22-Tetracosahexaene, 2,6,10,15,19,23-hexamethyl-, (all-E)- | NF | 22.567 | C30H50 | 2 |
| Cpd 99: 5-Hexyl-3,3-dimethyl-1-cyclopentene | NF | 22.754 | C13H24 | 10 |
| Cpd 100: (7E)-4,8,12-Trimethyltrideca-2,3,7,11,-tetraene-1-ol | NF | 22.917 | C16H26O | 3 |
| Cpd 101:(-)-Campherenone | Campherenone | 23.016 | C15H24O | 5 |
| Cpd 102: 5,8-methano-3,4,4a,5,6,7,8,8a-octahydro-1H-2-benzopyran-1-one | NF | 23.104 | C10H14O2 | 1 |
| Cpd 103: 1-(hydroxymethyl)-2-vinylcyclopentane | NF | 23.21 | C8H14O | 10 |
| Cpd 104: 3,4-Dimethylhexanedial | NF | 23.431 | C8H10O2 | 10 |
| Cpd 105: 4-Isopropenyl-4,7-dimethyl-1-oxaspiro[2.5]octane | NF | 24.037 | C12H20O | 10 |
| Cpd 106: 6-Methyl-2-heptanone | NF | 24.358 | C8H16O | 10 |
| Cpd 107: Bicyclo[4.3.0]nonane, 7-methylene-2,4,4-trimethyl-2-vinyl- | NF | 24.449 | C15H24 | 10 |
| Cpd 108: myrcene | Mycrene | 24.592 | C10H16 | 1 |
| Cpd 109: 5-Isopropyl-3-phenyl-2-thiohydantoin | NF | 24.776 | C12H14N2OS | 1 |
| Cpd 110: 4-[Acetylmethyl]-3,3-dimethyl-3,4-dihydrobenzopyran-2H-2-one | NF | 24.91 | C14H16O3 | 1 |
| Cpd 111: 15-Chloro-4-pentadecyne | NF | 25.483 | C15H27Cl | 10 |
| Cpd 112: longipinanol | longipinanol | 25.663 | C15H26O | 3 |
| Cpd 113: 1-Methyl-6-(3-methylbuta-1,3-dienyl)-7-oxabicyclo[4.1.0]heptane | NF | 25.831 | C12H18O | 10 |
| Cpd 1114: 3-Buten-2-ol, 3-methyl-4-(2,6,6-trimethyl-2-cyclohexen-1-yl)- | NF | 26.258 | C14H24O | 10 |
| Cpd 115: 7,7-dichlorobicyclo[3.2.0]hept-2-en-6-one | NF | 26.634 | C15H24O | 10 |
| Cpd 116: 2-Isopropyl-tricyclo[4.3.1.1(2,5)]undec-3-en-10-ol | NF | 26.819 | C14H22O | 10 |
| Cpd 117: But-3-enal, 2-methyl-4-(2,6,6-trimethyl-1-cyclohexenyl)- | NF | 27.177 | C14H22O | 10 |
| Cpd 118: 6,6,10-Trimethylundeca-3,8,10-triene-2,7-dione | NF | 27.3 | C14H20O2 | 3 |
| Cpd 119: 5-Iminopyrrolidine-2-carbonitrile | NF | 27.746 | C5H7N3 | 7 |
| Cpd 120: 7,7-dichlorobicyclo[3.2.0]hept-2-en-6-one | NF | 27.908 | C15H24O | 10 |
| Cpd 121: 1,3,5-Cycloheptatriene, 3,4-diethyl-7,7-dimethyl- | NF | 28.274 | C13H20 | 10 |
| Cpd 122: juniper camphor | Juniper camphor | 28.487 | C15H26O | 10 |
| Cpd 123: Longifolenaldehyde | longifolenaldehyde | 28.782 | C15H24O | 10 |
| Cpd 124: (E)-3-(3'-methyl-1',3'-butadienyl)-2,4,4-trimethylcyclohexanone | NF | 29.125 | C14H22O | 10 |
| Cpd 125: N-2-Methylpropanoyl-3,4-dihydro-1H-2,1-benzoxazine | NF | 29.524 | C12H15NO2 | 2 |
| Cpd 126: Benzo[e]isobenzofuran-1,4-dione,1,3,4,5,5a,6,7,8,9,9a-decahydro-6,6,9a-trime | NF | 29.9 | C15H20O3 | 10 |
| Cpd 127: (+-)cis-1,2-Dihydroxy-1,2,3,4-tetrahydrochrysene | NF | 29.922 | C18H16O2 | 5 |
| Cpd 128: 3h-Cyclodeca[B]Furan-2-One, 4,9-Dihydroxy-6-Methyl-3,10-Dimethylene-3a | NF | 30.391 | C15H20O4 | 10 |
| Cpd 129: Cyclopentanecarboxaldehyde, 2-methyl-3-methylene- | NF | 30.596 | C8H12O | 10 |
| Cpd 130: Cycloisolongifolene | Cycloisolongifolene | 31.629 | C15H24 | 10 |
| Cpd 131: aromadendrene 2 | aromadendrene | 31.933 | C15H24 | 10 |
| Cpd 132: Cyclopentanone, 2-(2-octenyl)- | NF | 32.24 | C13H22O | 10 |
| Cpd 133: iso-velleral | Isovelleral | 32.564 | C15H20O2 | 10 |
| Cpd 134: iso-velleral | Isovelleral | 32.907 | C15H20O2 | 10 |
| Cpd 135: allyl ionone 1 | Allyl Ionone | 33.71 | C16H24O | 10 |
| Cpd 136: Cyclohexane, (1-Bromo-3,3-dimethyl-2-hydroxybutylidene)- | NF | 34.152 | C12H21BrO | 6 |
| Cpd 137: Androstan-17-one, 3-ethyl-3-hydroxy-, (5.alpha.)- | Androstan | 34.34 | C21H34O2 | 10 |
| Cpd 138: Ethyl 5,8,11,14,17-eicosapentaenoate | NF | 35.817 | C22H34O2 | 10 |
| Cpd 139: 2-(4a,8-Dimethyl-1,2,3,4,4a,5,6,7-octahydro-naphthalen-2-yl)-prop-2-en-1-ol | NF | 36.021 | C15H24O | 10 |
| Cpd 140: 1,2-Benzenedicarboxylic acid, bis(2-ethylhexyl) ester | DEHP/ DNOP | 39.649 | C24H38O4 | 10 |
| Cpd 141: Cholest-5-ene, 3-bromo-, (3.beta.) | NF | 48.262 | C27H45Br | 10 |

NF: Not found
