# Supplementary material for: Comparative chemical profiling, cholinesterase inhibitions and anti-radicals properties of essential oils from Polygonum hydropiper L: A Preliminary anti- Alzheimer’s study
Source: Lipids Health Dis. 2015 Nov 4;14:141. doi: 10.1186/s12944-015-0145-8 (PMC4632677; doi:10.1186/s12944-015-0145-8)
Supplement: Additional file 2: Table S2. — Details of compounds identified in GC, GC-MS analysis of essential oils from flowers of Polygonum hydropiper. (DOCX 28 kb) [file 12944_2015_145_MOESM2_ESM.docx]

**Additional file 2: Table S2:** Detail of compounds identified in GC, GC-MS analysis of essential oils from flower of *P. hydropiper*.

| Compound Label | Common Name | RT | Formula | Hits (DB) |
| --- | --- | --- | --- | --- |
| Cpd 1: 1-Chlorooctylacetate | NF | 5.891 | C19H17ClO2 | 10 |
| Cpd 2: (1E)-1-(Pentyloxy)-1-butene | NF | 6.066 | C9H18O | 10 |
| Cpd 3:Cyclohexene, 1-methyl-4-(1-methylethenyl) | Nesol/ dl-Limonene | 6.355 | C10H16 | 10 |
| Cpd 4:1,1,3-Trimethyl-2-cyclohexanone | NF | 6.479 | C9H16O | 10 |
| Cpd 5:(2E)-4-Hydroxy-2-pentenoic acid | NF | 6.574 | C5H8O3 | 10 |
| Cpd 6: Pentanoic acid, 4-oxo | Levulinic acid | 6.751 | C5H8O3 | 10 |
| Cpd 7: n-Caproic acid vinyl ester | Vinyl caproate | 7.185 | C8H14O2 | 10 |
| Cpd 8: 1-Nonen-4-ol, 3,4-dimethyl | NF | 7.519 | C11H22O | 10 |
| Cpd 9: Hydroxylamine, O-hexyl | O-Hexylhydroxylamine | 7.852 | C6H15NO | 10 |
| Cpd 10: (Z/E)-1-Pentyl-1-hexenyl trifluoromethane sulfonate | NF | 7.94 | C12H21F3O3S | 10 |
| Cpd 11: Acetic acid, 1-methylcyclopentyl ester | 1-Methylcyclopentyl acetate | 8.042 | C8H14O2 | 10 |
| Cpd 12: Hexane, 1-(hexyloxy)-2-methyl | 1-(Hexyloxy)-2-methylhexane | 8.219 | C13H28O | 10 |
| Cpd 13: 2-Pentanone, 4-hydroxy-4-methyl | Tyranton/ Diacetone alcohol | 8.272 | C6H12O2 | 10 |
| Cpd 14: Cyclopropanemethanol, .alpha.-methyl-.alpha.-propyl- | NF | 8.362 | C8H16O | 10 |
| Cpd 15: Hexanal, 5-methyl- | 5-Methylhexanal | 8.781 | C7H14O | 10 |
| Cpd 16: Octanal, 7-hydroxy-3,7-dimethyl- | Fixol/Phixia | 8.977 | C10H20O2 | 10 |
| Cpd 17: Octanoic acid | Octylic acid/ Caprylic acid | 9.261 | C8H16O2 | 10 |
| Cpd 18: Ethanone, 1-(methylphenyl)- | Methyl tolyl | 9.839 | C9H10O | 10 |
| Cpd 19: Butanamide, 3-methyl- | Isovaleramide | 10.437 | C5H11NO | 10 |
| Cpd 20: Trifluoroacetic acid, 4-methylpentyl ester | Trifluoroacetate | 10.504 | C8H13F3O2 | 10 |
| Cpd 21: E and Z isomers of 2,6-Dimethyl-2,5-decadiene | NF | 11.118 | C12H22 | 10 |
| Cpd 22: 2-(1,1-Dimethylbutyl)oxirane | NF | 11.759 | C8H16O | 10 |
| Cpd 23: Myrcenyl acetate | Myrcenyl acetate | 11.999 | C12H20O2 | 10 |
| Cpd 24: 2-acetoxy-2-methyl-4-(2',6',6'-trimethylcyclohex -1'-en-1'-yl)trans-but-3-ene | NF | 12.997 | C16H26O2 | 10 |
| Cpd 25: Hydroxy-.alpha.-terpenyl acetate | NF | 13.6 | C12H20O3 | 2 |
| Cpd 26: Cyclopropanemethanol, 2-methyl-2-(4-methyl-3-pentenyl)- | NF | 13.939 | C11H20O | 10 |
| Cpd 27:CIS-1,3-Diisopropenyl-Trans-4-Vinyl-4-Methylcyclohexane | beta.-Elemene | 14.331 | C15H24 | 10 |
| Cpd 28: 2-Butanone, 4-(2,2-dimethyl-6-methylenecyclohexyl)- | NF | 14.437 | C13H22O | 10 |
| Cpd 29: 4-Caranol, (1S,3S,4R,6R)-(-)- | NF | 14.813 | C10H18O | 10 |
| Cpd 30:5,9-UNDECADIEN-2-ONE, 6,10-DIMETHYL-, (Z)- | Geranyl acetone ii | 15.484 | C13H22O | 10 |
| Cpd 31: Cyclopentane, 2-ethylidene-1,1-dimethyl- | NF | 15.619 | C9H16 | 10 |
| Cpd 32: 1-Isopropyl-3,4-dimethyl-1,3-cyclohexanediol | NF | 15.746 | C11H22O2 | 3 |
| Cpd 33: 9-Oxononanoic acid | 9-Oxononanoic acid | 16.031 | C9H16O3 | 10 |
| Cpd 34: Dehydrolinalool | Dehydrolinalool | 16.201 | C10H16O | 10 |
| Cpd 35: Cyclohexane, 2,4-diisopropenyl-1-methyl-1-vinyl-, (1S,2R,4R)- (-)- | NF | 16.367 | C15H24 | 10 |
| Cpd 36: 2(3H)-Benzofuranone, hexahydro-4,4,7a-trimethyl | Tetrahydroactinidiolide | 16.506 | C11H18O2 | 10 |
| Cpd 37: Limonene Dioxide 3 | Limonene Dioxide 3 | 16.75 | C10H16O2 | 10 |
| Cpd 38: [4aS,8aS] - 1,2,3,4,4a,5,8,8a - octahydro - 1,1,4a,6 - tetramethyl - 5 - met | NF | 16.821 | C15H24 | 10 |
| Cpd 39: 2(1H)-Naphthalenone, octahydro-4a,7,7-trimethyl-, cis- | NF | 17.177 | C13H22O | 10 |
| Cpd 40: Bicyclo[2.2.1]heptan-2-ol, 1,3,3-trimethyl-, acetate, (1S-exo)- | NF | 17.391 | C12H20O2 | 10 |
| Cpd 41:1,6-Octadien-3-ol, 3,7-dimethyl-, acetate | Bergamol | 17.391 | C12H20O2 | 10 |
| Cpd 42: 2(1H)-Naphthalenone, octahydro-4a,7,7-trimethyl-, trans- | NF | 17.569 | C13H22O | 10 |
| Cpd 43: 2H-Inden-2-one, 1,4,5,6,7,7a-hexahydro-4-methyl-7-(1-methylethyl) | NF | 17.715 | C13H20O | 10 |
| Cpd 44: Tripropyl - methylene - cyclohexadiene | NF | 17.911 | C16H26 | 1 |
| Cpd 45: 1-Cyclohexanol, 2-(3-methyl-1,3-butadienyl)-1,3,3-trimethyl- | NF | 17.97 | C14H24O | 10 |
| Cpd 46: 1,5-Cycloundecadiene, 8,8-dimethyl-9-methylene- | NF | 18.148 | C14H22 | 10 |
| Cpd 47: (-)-Caryophyllene oxide | beta.-Caryophyllene epoxide | 18.319 | C15H24O | 10 |
| Cpd 48: (1R,5S,8R,9R)-4,4,8-trimethyltricyclo[6.3.1.0(1,5)]dodeca-2-en-9-ol | NF | 18.374 | C15H24O | 10 |
| Cpd 49:2,5,5,6,8a-Pentamethyl-trans-4a,5,6,7,8,8a-hexahydro-gamma-chromene | NF | 18.532 | C14H24O | 10 |
| Cpd 50: 3,5-Diisopropenyl-1,1,2-trimethylcyclohexane | NF | 18.611 | C15H26 | 10 |
| Cpd 51:2-Undecynoic acid, ethyl ester | Ethyl decyne carbonate | 18.735 | C13H22O2 | 10 |
| Cpd 52: Humulene Oxide | Humulene Oxide | 18.82 | C15H24O | 10 |
| Cpd 53: Cyclohexane, 1,1-dimethyl-2,4-bis(1-methylethenyl)-, cis- | NF | 19.078 | C14H24 | 10 |
| Cpd 54: 1-Oxaspiro[2.5]octane, 5,5-dimethyl-4-(3-methyl-1,3-butadienyl)- | NF | 19.206 | C14H22O | 10 |
| Cpd 55:(3Z)-3-Heptadecen-5-yne | NF | 19.315 | C17H30 | 10 |
| Cpd 56: 2-Isopropenyl-5-acetyl-4-hydroxy-2,3-dihydrobenzofuran | NF | 19.365 | C13H14O3 | 10 |
| Cpd 57:2,6,10-Dodecatrien-1-ol, 3,7,11-trimethyl- | Farnesol | 19.474 | C15H26O | 10 |
| Cpd 58: Selina-6-en-4-ol | NF | 19.67 | C15H26O | 10 |
| Cpd 59: trans-4-Methyl-2-penten-1-ol | NF | 19.88 | C6H12O | 1 |
| Cpd 60: (-)-Caryophyllene oxide | beta.-Caryophyllene epoxide | 19.969 | C15H24O | 10 |
| Cpd 61: (-)-Caryophyllene oxide | beta.-Caryophyllene epoxide | 20.049 | C15H24O | 10 |
| Cpd 62: 4-Isopropenyl-4,7-dimethyl-1-oxaspiro[2.5]octane | NF | 20.145 | C12H20O | 10 |
| Cpd 63:1-Hexyl-1-cyclohexene | NF | 20.235 | C12H22 | 10 |
| Cpd 64: Z-limonene-1,2-epoxide | Trans-Limonenoxide | 20.285 | C10H16O | 10 |
| Cpd 65: 3-Buten-2-ol, 2-methyl-4-(1,3,3-trimethyl-7-oxabicyclo[4.1.0]hept-2-yl)- | NF | 20.367 | C14H24O2 | 10 |
| Cpd 66: 1,3,5-Pentanetricarboxamide, N,N',N''-triheptyl-N,N',N''-trimethyl- | NF | 20.582 | C12H20O | 10 |
| Cpd 67: 1,2-Di[(2e)-2-Butenyl]Cyclohexane | NF | 20.719 | C14H24 | 6 |
| Cpd 68: 1-Phenylnonane | Nonylbenzene | 20.821 | C15H24 | 10 |
| Cpd 69: Cyclopentane, 2-(1,1-dicyanomethyl)-1-isopropenyl-3-methyl- | NF | 21.014 | C12H16N2 | 1 |
| Cpd 70: (1R,2S,4R,8S)-1,2:8,9-Diepoxy-p-menthane | NF | 21.19 | C10H16O2 | 10 |
| Cpd 71: Cycloisolongifolene | Cycloisolongifolene | 21.272 | C15H24 | 10 |
| Cpd 72: Caryophyllene oxide | Caryophylene oxide | 21.432 | C15H24O | 10 |
| Cpd 73: 2-Acetonyl cycloheptanone(3-.gamma.) | NF | 21.519 | C10H16O2 | 10 |
| Cpd 74: Caryophyllene oxide | Caryophylene oxide | 21.763 | C15H24O | 10 |
| Cpd 75: 1,1,4,7-Tetramethyldecahydro-1h-Cyclopropa[E]Azulen-4-Ol | Globulol | 21.943 | C15H26O | 10 |
| Cpd 76: (-)-DRIM-7-EN-11-OL | Drimenol | 22.167 | C15H23O | 10 |
| Cpd 77: 2-Butanol, 4-[2,2,6-trimethylcyclohexyl]-, acetate | Tetrahydroionyl acetate | 22.38 | C15H28O2 | 10 |
| Cpd 78: (2.alpha.,4a.beta.,8.beta.,8a.beta.)-(+-)1-(Decahydro-8-hydroxy-4a,8-dimethy | NF | 22.654 | C14H24O2 | 4 |
| Cpd 79: 2,6,10-Dodecatrien-1-ol, 3,7,11-trimethyl- | Farnesol | 22.73 | C15H26O | 10 |
| Cpd 80: 4-Hydroxy-9-methyltetracyclo[6.4.2.0(1,9).0(4,14)]tetradeca-3,13-dione | NF | 22.876 | C15H20O3 | 1 |
| Cpd 81: 6,11-Undecadiene, 1-acetoxy-3,7-dimethyl- | NF | 23.095 | C16H28O2 | 10 |
| Cpd 82: 7-(1,3-dimethylbuta-1,3-dienyl)-1,6,6-trimethyl-3,8-dioxatricyclo[5.1.0.0(2,... | NF | 23.121 | C15H22O2 | 10 |
| Cpd 83: cis-Z-.alpha.-Bisabolene epoxide | Bisabolene epoxide | 23.452 | C15H24O | 10 |
| Cpd 84: Caryophyllene oxide | Caryophylene oxide | 23.656 | C15H24O | 10 |
| Cpd 85: Geranyl acetate, 2,3-epoxy- | NF | 23.795 | C12H20O3 | 6 |
| Cpd 86: 2(1H)-Naphthalenone, octahydro-4a,7,7-trimethyl-, cis- | NF | 23.989 | C13H22O | 10 |
| Cpd 87: 2-Heptanone, 6-methyl- | 6-Methyl-2-heptanone | 24.222 | C8H16O | 10 |
| Cpd 88: Nerolidol-Epoxyacetate | Nerolidol-Epoxyacetate | 24.391 | C17H28O4 | 10 |
| Cpd 89: 2-Oxabicyclo[2.2.2]octane, 1,3,3-trimethyl- | Eucalyptol | 24.565 | C10H18O | 10 |
| Cpd 90: Allyl ionone 4 | Allyl ionone 4 | 24.719 | C16H24O | 10 |
| Cpd 91: Cyclopropane, 1-(1-hydroxy-1-heptyl)-2-methylene-3-pentyl- | NF | 24.834 | C16H30O | 1 |
| Cpd 92: cis-Z-.alpha.-Bisabolene epoxide | Bisabolene epoxide | 25.117 | C15H24O | 10 |
| Cpd 93: 4(1H)-Azulenone, octahydro-1-methylene-, trans- | NF | 25.469 | C11H16O | 10 |
| Cpd 94: Cedranoxide, 8,14- | Cedranoxide | 25.913 | C15H24O | 10 |
| Cpd 95: (-)-Sinularene | Sinularene | 26.026 | C15H24 | 10 |
| Cpd 96: Spiro[2.5]octane, 3,3-dimethyl-2-(1-buten-3-on-1-yl)- | NF | 26.585 | C14H22O | 10 |
| Cpd 97: cis-Z-.alpha.-Bisabolene epoxide | alpha.-Bisabolene epoxide | 26.765 | C15H24O | 10 |
| Cpd 98: (4E)-5,9-Dimethyl-4,8-decadien-3-ol | NF | 27.011 | C12H22O | 5 |
| Cpd 99: Menthol, 1'-(butyn-3-one-1-yl)-, (1R,2S,5R)- | Menthol | 27.164 | C14H22O2 | 10 |
| Cpd 100: Aristolen epoxide | Aristolen epoxide | 27.675 | C15H24O | 10 |
| Cpd 101: Hexadecanoic acid /n-Hexadecoic acid | Palmitic acid | 27.796 | C16H32O2 | 10 |
| Cpd 102: cis-5,8,11,14,17-Eicosapentaenoic acid, methyl ester | NF | 27.939 | C21H32O2 | 10 |
| Cpd 103: Iso-Velleral | Isovelleral | 28.12 | C15H20O2 | 10 |
| Cpd 104:(+)-Ledol | Ledol | 28.28 | C15H26O | 10 |
| Cpd 105: Citronellyl Valerate | Citronellyl Valerate | 28.59 | C15H28O2 | 1 |
| Cpd 106: 2,4a,8,8-Tetramethyldecahydrocyclopropa[d]naphthalene | NF | 29.078 | C15H26 | 10 |
| Cpd 107: 7-isopropenyl-1,4a-dimethyl-4,4a,5,6,7,8-hexahydro-2(3h) -naphthalenone | NF | 29.387 | C15H21DO | 10 |
| Cpd 108: Benzo[e]isobenzofuran-1,4-dione,1,3,4,5,5a,6,7,8,9,9a-decahydro-6,6,9a-trime | NF | 29.542 | C15H20O3 | 10 |
| Cpd 109: 3h-cyclodeca[b]furan-2-one, 4,9-dihydroxy-6-methyl-3,10-Dimethylene-a,4,7,8 | NF | 30.091 | C15H20O4 | 10 |
| Cpd 110: Benzene, 1,1'-bis(1-hexene-3-one-4-methyl-1,6-diyl) | NF | 30.293 | C19H20O | 1 |
| Cpd 111: DRIMINOL | Drimenol | 30.409 | C15H26O | 10 |
| Cpd 112: (Z)-3-Butylidene-4,5-methylenedioxyphthalide | NF | 30.948 | C13H12O4 | 8 |
| Cpd 113: cis-Z-.alpha.-Bisabolene epoxide | Bisabolene epoxide | 31.018 | C15H24O | 10 |
| Cpd 114: Longifolene-(I2)-epoxide-(1) | Longifolene epoxide | 31.356 | C15H24O | 10 |
| Cpd 115: ISO-VELLERAL | Isovelleral | 32.24 | C15H20O2 | 10 |
| Cpd 116: cis-Z-.alpha.-Bisabolene epoxide | Bisabolene epoxide | 33.615 | C15H24O | 10 |
| Cpd 117: 2h-cyclopropa[g]benzofuran, 4,5,5a,6,6a,6b-hexahydro-4,4,6b-Trimethyl-2-(1- | NF | 33.786 | C15H22O | 10 |
| Cpd 118: 7-isopropenyl-1,4a-dimethyl-4,4a,5,6,7,8-hexahydro-2(3h)-Naphthalenone | NF | 34.065 | C15H21DO | 10 |
| Cpd 119: Arteannuin b | Arteannuin-b | 35.544 | C15H20O3 | 10 |
| Cpd 120: 2-(4a,8-Dimethyl-1,2,3,4,4a,5,6,7-octahydro-naphthalen-2-yl)-prop-2-en-1-ol | NF | 35.717 | C15H24O | 10 |
| Cpd 121: 3,4-Dihydro-3,3,4,4-tetramethyl-1H-[2]benzazepine-1,5(2H)-dione | NF | 43.7 | C14H17NO2 | 4 |
| Cpd 122: N-tert-Butylmaleimide | N-Tert-Butylmalimide | 58.257 | C8H11NO2 | 10 |

**NF:** Not found
